# Remote Sensing of Ecology, Biodiversity and Conservation: A Review from the Perspective of Remote Sensing Specialists

**DOI:** 10.3390/s101109647

**Published:** 2010-11-01

**Authors:** Kai Wang, Steven E. Franklin, Xulin Guo, Marc Cattet

**Affiliations:** 1 Department of Geography and Planning, University of Saskatchewan, Saskatoon, SK S7N 5C8, Canada; E-Mail: xulin.guo@usask.ca; 2 Trent University, Peterborough, ON K9J 7B8, Canada; E-Mail: sfranklin@trentu.ca; 3 Department of Veterinary Pathology, University of Saskatchewan, Saskatoon, SK S7N 5B4, Canada; E-Mail: marc.cattet@usask.ca

**Keywords:** remote sensing, EBC (ecology, biodiversity and conservation), thermal infrared, small-satellite constellation, LIDAR, image classification, data fusion, integration of remote sensing (RS) and geographic information system (GIS)

## Abstract

Remote sensing, the science of obtaining information via noncontact recording, has swept the fields of ecology, biodiversity and conservation (EBC). Several quality review papers have contributed to this field. However, these papers often discuss the issues from the standpoint of an ecologist or a biodiversity specialist. This review focuses on the spaceborne remote sensing of EBC from the perspective of remote sensing specialists, *i.e.*, it is organized in the context of state-of-the-art remote sensing technology, including instruments and techniques. Herein, the instruments to be discussed consist of high spatial resolution, hyperspectral, thermal infrared, small-satellite constellation, and LIDAR sensors; and the techniques refer to image classification, vegetation index (VI), inversion algorithm, data fusion, and the integration of remote sensing (RS) and geographic information system (GIS).

## 1. Introduction

In general, ecological research refers to the investigation of organisms and their surrounding environment, including biotic and abiotic entities. Due to the multifaceted nature of biodiversity, it is difficult to simply express and measure biodiversity. Biodiversity should be related to not only the variation of life forms, but also the ecological complexes of which they are a part. Conservation has become an indispensable way of dealing with the accelerated native ecosystem conversion and degradation, which have a significantly negative effect on biodiversity. Remote sensing, the science of obtaining information via noncontact recording [1], has swept the fields of ecology, biodiversity and conservation (EBC). Remote sensing can provide consistent long-term Earth observation data at scales from the local to the global domain. In addition, remote sensing is not labor-intensive and time-consuming, compared with field-based observations. The review papers of Kerr and Ostrovsky and Turner *et al.*, published in the journal of “*Trends in Ecology and Evolution*”, has been cited hundreds of times by scientists from around the world who are involved in remote sensing of EBC [2,3]. Turner *et al.* stated two categories of approaches, namely direct and indirect remote sensing approaches [3]. The direct approach refers to the direct observation of individual organisms, species assemblages, or ecological communities from airborne or satellite sensors, such as the application of high spatial resolution and hyperspectral sensors (e.g., [4]). Indirect approaches rely on environmental parameters derived from remotely sensed data as proxies. For example, habitat parameters, such as land cover, species composition, *etc.*, can be considered as a surrogate for precise estimates of potential species ranges and patterns of species richness [5]. The Foothills Research Institute Grizzly Bear Program (FRIGBP, formerly called Foothills Model Forest Grizzly Bear Research Program) has successfully applied this kind of approach in west-central Alberta (Canada) [6]. Kerr and Ostrovsky described ecological remote sensing in three main areas [2]. First, land cover classification, the physiographical characteristics of the surface environment, can be used to identify very specific habitats and predict the distribution of both individual species and species assemblages at a large spatial extent (e.g., [7]). Secondly, integrated ecosystem measurements offer the urgently needed measurements of functions at different spatial scales, including whole ecosystems, such as the derivation of leaf area index (LAI) and net primary productivity (NPP) mostly based on the normalized difference vegetation index (NDVI, e.g., [8]). Thirdly, change detection provides near-continuous, long-term measurements of key ecological parameters in order to monitor ecosystem through time and over significant areas, such as the application of climate change and habitat loss (e.g., [9]). Additionally, several quality review papers have contributed to this field, such as [10–14].

Most existing review papers too often discuss an issue from the viewpoint of ecologists or biodiversity specialists. For instance, Aplin reviewed the remote sensing of ecology as it relates to the significance of remote sensing in ecology, to spatial scale, and to terrestrial and marine ecological applications [11]. Gillespie *et al.* discussed the development of measuring and modeling biodiversity from space with a focus on species and land-cover classifications, modeling biodiversity, and conservation planning [14]. This review, on the other hand, focuses on the spaceborne remote sensing of EBC from the perspective of remote sensing specialists, *i.e.*, it is organized in the context of state-of-the-art remote sensing technology, including instruments and techniques. Herein, the instruments to be discussed consist of high spatial resolution, hyperspectral, thermal infrared, small-satellite constellation, and LIDAR sensors; and the techniques refer to image classification, vegetation index (VI), inversion algorithm, data fusion, and the integration of remote sensing (RS) and geographic information system (GIS).

## 2. Advanced Instruments in Remote Sensing of EBC

Based on the current status of remote sensing instruments, their existing applications in the literature, and future potential contributions to EBC, the aforementioned five types of instruments: high spatial resolution, hyperspectral, thermal infrared, small-satellite constellation, and LIDAR sensors, were selected. In order to avoid overlapping between high spatial resolution and hyperspectral sensors, the hyperspectral sensors discussed below will mainly refer to sensors with medium spatial resolution, such as Hyperion with 30 m spatial resolution. Radar sensors are not selected because their applications mostly concentrate on geology, ice and snow, marine surveillance, and agriculture. In addition, some uncertainties in radar remote sensing, such as the saturation issue under high vegetation biomass, hamper its applications on EBC.

### 2.1. High Spatial Resolution

Generally speaking, high spatial resolution, also called fine spatial resolution, is less than 10 m, and ranges from 0.5–10 m in the commercial domain for environmental research. IKONOS, QuickBird, OrbView-3 and SPOT-5 (Satellite Pour l’Observation de la Terre-5) are the commonly used systems (see [15] for the high-spatial resolution optical sensors). The benefit of high spatial resolution imagery is that it greatly increases the accuracy of identification and characterization of small objects at spatial scales which were previously only available from airborne platforms [3,14]. For example, Gillespie *et al.* provided several examples of accurately identifying plant species based on the high spatial resolution imagery [14]. Turner *et al.* have pointed out it is applicable and feasible to directly identify certain species and species assemblages at the scale of high spatial resolution [3]. In addition, high spatial resolution imagery can be employed to assess the accuracy of remote sensing precuts derived from moderate or coarse spatial resolution imagery. For instance, Wabnitz *et al.* assessed the accuracy of Landsat-based large-scale seagrass mapping against patterns detectable with very high-resolution IKONOS images [16]. However, the high spatial resolution imagery is still expensive to acquire from commercial satellites, at the price of approximately 3,000–5,000 US$ for 10 km^2^ [14], although it has tended to decrease with the emergence of more sensors and the upcoming competition. Moreover, data coverage and security restrictions are still a significant hurdle before easily accessing high spatial resolution satellite data [17].

Due to the large amount of high spatial resolution sensors, the commonly-used IKONOS imagery was selected to display their typical applications in 2008 and 2009. First of all land cover, as the representative of basic landscape information, can be extracted quickly and reliably based on the high spatial resolution data. For example, the object-oriented classification of IKONOS-2 satellite images was utilized to explicitly recognize the transitional areas between tree crowns and tree shades (tree shadows), and then for the quantification of canopy cover [18]. Further, IKONOS imagery can be used to quantify and evaluate the spatial structure of critical habitats and how it affects endemic species, which is essential baseline information for biodiversity monitoring and management (e.g., [19]). In the context of marine applications, areas of coastline, with their fertile soil and unique flora and fauna which need to be highly protected, were planned for in a sustainable way through mapping the changes in land use of the area based on IKONOS imagery in the Cesme Peninsula (Turkey) [20]. Improving the science and conservation of coral reef ecosystems, such as the significant fish-habitat relationship, is often the objective of marine ecology, and also is an important facet in the application of IKONOS imagery [21]. Harborne *et al.* examined intra-habitat variability in coral-reef fish by mapping habitat heterogeneity, which is always considered a surrogate of biodiversity, in order to aid the design of networks of marine reserves [22]. Although high spatial resolution satellite remote sensing has been hailed as a very useful source of data, Nagendra and Rocchini pointed out that high spatial resolution remotely sensed data are one of the most potentially powerfully yet underutilized sources for tropical research on biodiversity, and stimulating discussion on the applications should be the first step in promoting a more extensive use of such data [17].

### 2.2. Hyperspectral

Hyperspectral data have the ability to collect ample spectral information across a continuous spectrum generally with 100 or more contiguous spectral bands. It is different from multispectral sensors which detect relatively few discrete bands [17]. Hundreds of spectral bands with 10–20 nm spectral bandwidths offer new possibilities to detect subtle differences between objects of interest. The best example is to discriminate fine-scale, species-specific land cover [3], such as vegetation categories or soil types [11], which make remarkable contribution to the study regarding biodiversity patterns. Moreover, Nagendra and Rocchini summarized that hyperspectral data have been successfully applied in recording information regarding critical plant properties (e.g., leaf pigment, water content and chemical composition), discriminating tree species in landscapes, and fairly accurate identification between different species [17]. What is more, spectral signatures acquired from atmosphere-corrected hyperspectral data can be directly compared to the existing spectral library (e.g., the Jet Propulsion Laboratory Spectral Library) in order to rapidly identify ground information useful in land-cover classification, characterization and change detection [3]. Similar to the situation with high spatial resolution imagery, the hyperspectral imagery encountered the same underutilization, and a high cost which may put it out of research for many ecologists [14], especially those in developing countries who eagerly need the data [17].

Shippert listed the existing hyperspectral sensors acquiring imagery from space, including the Hyperion sensor on NASA’s EO-1 (National Aeronautics and Space Administration’s Earth Observing-1), the CHRIS (Compact High Resolution Imaging Spectrometer) sensor on the European Space Agency’s PROBA (PRoject for On-Board Autonomy) satellite, and the FTHSI (Fourier Transform Hyperspectral Imager) sensor on the U.S. Air Force Research Lab’s MightySat II satellite [23]. Of these sensors, the first-civilian and most commonly used data are derived from the Hyperion, which is operated by the EROS (Earth Resources Observation and Science) at a relatively low cost to the general public [23]. The EO-1, on which the Hyperion sensor is, was launched in November, 2000 as a one-year technology validation and demonstration in support of the LDCM (Landsat Data Continuity Mission; [24]). The Hyperion sensor, an upgrade from the LEWIS Hyperspectral Imaging Instrument (HSI), records visible light and other reflected electromagnetic energy in 220 spectral bands from 0.4 to 2.5 μm at a 30 m resolution [25]. Table 1 lists the Hyperion characteristics.

The recent applications of Hyperion hyperspectral imagery mainly include ecology and biodiversity in forest, grassland [27], agriculture [28], and vegetation [29], fragmented ecosystem and ecosystem succession, coastal environment [30], *etc.* For example, vegetation types and densities were classified in support of the wildfire management, that is, fire propagation simulation models and fire risk assessment were based on a Hyperion classification map with 93% accuracy [31]. Foster *et al.* proposed hyperspectral imagery from EO-1 Hyperion is capable of mapping low-lying woody lianas, which are critical to tropical forest dynamics because of their strong influence on forest regeneration, disturbance ecology, and biodiversity [32]. Pignatti *et al.* analyzed the capability of Hyperion data for discriminating land cover in a complex natural ecosystem according to the structure of the currently used European standard classification system (CORINE Land Cover 2000), and the results showed the potential of the imagery up to the 4th level of the CORINE legend, even at the sub-pixel level, within a fragmented ecosystem [33]. Besides the application of land cover classification, the relationships between LAI and spectral reflectance were studied by [34] using narrowband (EO-1 Hyperion) and broadband (Landsat ETM+ [Enhanced Thematic Mapper Plus]) remotely sensed data in Sulawesi (Indonesia). Nagendra and Rocchini preliminarily discussed the strengths and drawbacks of hyperspatial (*i.e.*, high spatial resolution) and hyperspectral data [17]. Hyperspatial data was considered to be best suited for facilitating the accurate location of features such as tree canopies, but less suited to the identification of aspects such as species identity. However, conversely, hyperspectral data appear capable of identifying features with significantly increasing accuracy. Therefore, the integration of Hyperion and IKONOS imagery was proposed to differentiate the subtle spectral differences of land-use/land-cover types on household farms in the Northern Ecuadorian Amazon with an emphasis on secondary and successional forests, and the promising results supported the integrated use of hyperspectral and hyperspatial data [35].

### 2.3. Thermal Remote Sensing

Thermal remote sensing detects the energy emitted from the Earth’s surface in the thermal infrared (TIR, 3 μm to 15 μm), which can be radiated by all bodies above absolute zero. Theoretically, TIR sensors measure the surface temperature and thermal properties of targets [36], which are essential for developing a better understanding, and more robust models, of land-surface energy balance interactions [37]. Moreover, TIR remote sensing is capable of uncovering the principles of ecological patterns of structure and function due to the development of ecological thermodynamics [37]. A thermal grey level image is generated based on relative radiant temperatures (a thermogram), and light tones correspond to warmer temperatures and dark tones to cooler temperatures [36]. TIR remote sensing plays an important role in observation of Earth surface characteristics, and is very useful for research regarding analysis of biophysical Earth processes, in particular landscape characterization and measurement of land surface processes [37]. The well-known sensors with TIR bands include the Advanced Very High Resolution Radiometer (AVHRR) onboard the Polar Orbiting Environmental Satellites (POES), the Landsat Thematic Mapper (TM) and ETM+, the Advanced Spaceborne Thermal Emission and Reflection Radiometer (ASTER) on the Terra Earth observing satellite platform, *etc.* [37].

TIR remote sensing has been developing since 1880, and has proven to be an integral part of understanding landscape characteristics [37], although it is relatively rarely used by ecologists [2]. However, interests have increasingly focused on the use of TIR remote sensing in EBC. For instance, biophysical variables were derived from thermal and multispectral remote sensing data and coupled with a Soil-Vegetation-Atmosphere-Transfer (SVAT) model [38]. Duro *et al.* pointed out the TIR region is an important source of information to study environmental disturbance because of the negative relationship between vegetation density and land surface temperatures [13]. Mildrexler *et al.* proposed a disturbance detection index using Moderate Resolution Imaging Spectroradiometer (MODIS) 16-day Enhanced Vegetation Index (EVI) and 8-day Land Surface Temperature (LST), and it was successfully applied to detect continental-scale disturbance events such as wildfire, irrigated vegetation, precipitation variability, and the incremental process of recovery of disturbed landscapes [39]. Another good use of TIR remote sensing data is to measure evapotranspiration, evaporation, and soil moisture. For example, Crow and Zhan analyzed the continental-scale performance of surface soil moisture retrieval algorithms depending on satellite passive microwave, scatterometer, and thermal remote sensing observations [40]. Petropoulos *et al.* reviewed Ts/VI (surface temperature/vegetation index) remote sensing based methods for the retrieval of land surface energy fluxes and soil surface moisture, and suggested one piece of future work should evaluate the accuracy of these methods under diverse environments [41].

### 2.4. Constellation of Small Satellites

A small satellite generally refers to its mass in the range of 1–500 kg and satellite constellation is defined as groups of satellites working in concert [42]. Since 1997, six symposia on small satellites have been organized by the International Academy of Astronautics (IAA) in Berlin, Germany. Kramer and Cracknell reviewed the development of small satellites in remote sensing [43]. With the launch of DMC (Disaster Monitoring Constellation, Table 2), the concept of the Earth-observation constellation of low-cost small satellites has been put into action. It is capable of obtaining multispectral images of any part of the world every day [24]. The DMC was initially proposed in 1996 and led by SSTL (Surrey Satellite Technology Limited), which is a world leader in high performance small satellites [42]. Wang *et al.* briefly introduced the characteristics of DMC imagery and its potential applications in environmental science [44]. Also, HJ-1 (Huan Jing-1, also called Environment-1, operated by China) is another outstanding constellation system. It is designed mainly for environmental protection and disaster monitoring, and the payload instruments onboard consist of a CCD (Charge-Coupled Device) camera, an infrared camera, a hyperspectral imager and an S-band SAR (Synthetic Aperture Radar, [45]).

Besides the benefits in cost and operation, the constellation of small satellites has two obvious advantages in applications, *i.e.*, global surveying and increased revisit frequency [24]. It is relatively easy to obtain observation data across the world in a short time for constellation systems. The increased revisit frequency can not only satisfy the application of detecting rapid surface changes such as crop-growth monitoring and detecting intraseasonal ecosystem disturbance, but also promotes acquisition of good-quality imagery with limited cloud-contamination. Wang *et al.* discussed the issue of clouds and cloud shadows in the environmental remote sensing community, and advised looking for good solutions to the unavoidable problem in optical remote sensing [44]. The development of a constellation of low-cost small satellites is believed to make contributions to this issue at the sensor level. Only a few studies of EBC applied the imagery of small-satellite constellation, though Aplin has predicted the bright future of this kind of satellite imagery [11]. Qian *et al.* demonstrated that simulated HJ-1B satellite data performed better on smaller and cooler fires than MODIS or AVHRR data, and believe it will offer a great opportunity for fire detection [46]. The FRIGBP has started testing the applicability of DMC imagery for wildlife large-area habitat mapping in west-central Alberta (Canada) [44].

### 2.5. LIDAR

Light Detection and Ranging (LIDAR), also called Laser altimetry, is an active remote sensing technology that utilizes a laser to illuminate a target object and a photodiode to register the backscatter radiation [47,48]. The current LIDAR remote sensing can be categorized into two general groups: non-scanning LIDARs, and scanning LIDARs. The non-scanning LIDARs record pulsed ranging that measures the travel time between the transmitted and received signal backscattered from the object surface, and the scanning LIDARs register continuous wave ranging that is produced in a transmitted sinusoidal signal and carried out by modulating the laser light intensity [49]. According to the characteristics of LIDAR technology, it has been proven to provide horizontal and vertical information at high spatial resolutions and vertical accuracies [47]. For example, Miller stated that 5–30 cm range is the typical accuracies for LIDAR-derived vertical information [50]. Airborne LIDAR remote sensing systems such as LVIS (Laser Vegetation Imaging Sensor) have been used for bathymetry, forestry, and other applications [48,51,52]. For instance, Turner *et al.* briefly discussed the airborne LIDAR remote sensing for biodiversity science and conservation [3]; Lim *et al.* reviewed the application within forest structure (vertical information) [47], e.g., canopy and tree height, biomass, and volume; Goetz *et al.* claimed species distribution models have been improved through airborne LIDAR quantifying vegetation structure within a landscape [53]. LIDAR was underlined by [11] as one of the strong interests of the remote sensing community in ecology. Besides airborne LIDAR with the limitations of large data volumes, footprint size and high costs [54], spaceborne LIDAR has come through with the launch of the ICESat/GLAS (Ice, Cloud, and Land Elevation Satellite/Geoscience Laser Altimeter System), which is the first laser-ranging instrument for continuous global observations. The applications of the GLAS data in EBC, which are seldom reviewed, will be discussed below.

LIDAR focused on the forest vertical structure, especially forest canopy height and aboveground biomass estimation. Lefsky *et al.* estimated forest canopy height with an RMSE of 5 m (83% of variance explained) in varied forest types including evergreen needle leaf, deciduous broadleaf and mixed stands in temperate North America, and tropical evergreen broadleaf forests in Brazil [55]. Mangrove forests are considered as one of the most biodiverse and productive wetlands on Earth, and the mangrove height and aboveground biomass were measured and mapped based on SRTM (Shuttle Radar Topography Mission) elevation data, GLAS waveforms and field data [56]. Pflugmacher *et al.* compared GLAS height and biomass estimates with reference data from the Forest Inventory and Analysis (FIA) program of the U.S. Forest Service at a regional scale, and promising results were obtained [57]. Helmer *et al.* proposed the combination of Landsat time series and the GLAS to estimate the biomass accumulation of the Amazonian secondary forest, and the estimation agreed well with ground-based studies [58]. Duncanson *et al.* tested simulated GLAS data under tough conditions, e.g., areas with dense forests, high relief, or heterogeneous vegetation cover, and demonstrated the capability of GLAS waveforms as supplemental model input to improve estimates of canopy height [54].

## 3. Advanced Techniques in Remote Sensing of EBC

Similar criteria were applied to choose the remote sensing techniques discussed below, including promising algorithms or methods in image classification, vegetation index (VI), inversion algorithm, data fusion, and the integration of RS and GIS. Although these techniques are reviewed separately, they are frequently integrated in practice. For example, data fusion can be implemented to remotely sensed data before they are classified by advanced classifiers in order to improve classification accuracy.

### 3.1. Image Classification

Regardless of the variety of uses for remote sensing images, the first goal is to extract landscape information from the satellite images [59]. Image classification has been recognized as the most effective means to do so since mid-1800s, when humans first identified different types of land-use and land-cover in aerial photography [60]. Jensen discussed in detail the fundamental elements of image interpretation including grayscale tone, color, height and depth, size, shape, texture, pattern shadow, site, association and arrangement [1]. With the widespread of digital computers, special-purpose image classification algorithms have been used to extract land-use/land-cover and biophysical information directly from remotely sensed data [60]. In order to derive more accurate classifications, new approaches have increasingly emerged, and such approaches have made significant contributions to the science of EBC, examples of which would be support vector machines (SVMs), one-class classifier, object-oriented classification, and fuzzy classifications.

SVMs consist of many theoretically superior machine learning algorithms, and make use of optimization algorithms to find an optimal separating hyperplane (OSH) between classes based on training samples [61]. The hyperplane is called support vectors [62]. Foody and Mathur have demonstrated the robustness of SVMs through comparison with artificial neural networks (ANNs) and machine learning decision trees, especially for small training sets [63]. The SVM was selected by [64] to help understand the relationships among spectral resolution, classifier complexity, and classification accuracy obtained with hyperspectral sensors for the classification of forest areas. Ichii *et al.* applied SVM-based evapotranspiration estimation to refine rooting depths for ecosystem modeling in California [65].

Commonly, only one specific class is the foci of research interest [66]. Due to the fact that conventional multiclass classifier may be suboptimal in terms of the classification accuracy of the class of interest, a one-class-classification approach was suggested to focus tightly on the specific class. For example, Sanchez-Hernandez *et al.* applied the one-class classifier based on the support-vector data description (SVDD) to map fenland habitat in support of conservation activities [66]. An accuracy of 97.5% and 93.6% from the user’s and producer’s perspectives was obtained, and it performed much better than conventional maximum-likelihood classification. In the same year, the classifier was used to map and monitor coastal saltmarsh habitats of high conservation value under the European Union’s Habitats Directive [67].

With the wide availability of high-spatial resolution satellite data, pixel-based classification algorithms seem not to be ideal to extract information desired from the data exhibiting high frequency components with high contrast and horizontal layover of objects [60]. Therefore, object-oriented classification algorithms have been developed to meet this need, and have established improved classification accuracy when compared with the traditional methods [5,60]. The basic processing units of object-oriented classification are segments, so-called image objects that represent a relatively homogenous unit on the ground [68]. Then classification was performed on image objects, and not on pixels. One of the most popular algorithms was developed to the software of Definiens’ Developer (also called eCognition; [69]). Advantages of object-oriented classification are to make full use of meaningful statistic and texture calculation, uncorrelated shape information (e.g., length-to-width ratio, direction and area of an object, *etc.*) and topological features (neighbor, super-object, *etc.*), and the close relation between real-world objects and image objects [68]. Jensen *et al.* pointed out that the advantages include rapid process, and scale flexibility in which users can select different scale levels according to their images [60]. A variety of studies applied object-oriented classification into the science of EBC. For example, Collingwood *et al.* classified agricultural areas in Alberta grizzly bear habitats based on one of the object-oriented classification techniques – sequential supervised masking (SSM), in order to help ecologists understand the relationship between crop types and grizzly bear presence [5]. Wang *et al.* proposed that object-oriented classification may traverse the possible Landsat-gap on applications such as landscape pattern analysis or ecological models [44].

Traditionally, land cover information is assigned into a finite number of non-overlapping classes, and the classes are mutually exclusive [70], which is described as the one-entity-one-class method [71]. However, pixels may contain more than one class because of the heterogeneity and the limitation in spatial resolution of remotely sensed data, especially in medium and coarse spatial resolution imagery [70]. And the presence of mixed pixels could not be removed totally no matter how accurately map classes are defined [71]. Therefore, fuzzy classification, also called subpixel classification, arose in the context of the uncertainty associated to class mixtures. In fuzzy systems, every pixel is supposed to consist of multiple and partial memberships of all candidate classes [70]. Spectral mixture analysis (SMA) is one of the most popular and most effective approaches for dealing with mixed pixel problem [60,70]. For example, Lu and Weng used linear SMA to explore the relationship between urban thermal features and biophysical descriptors based on ASTER images [72]. Plourde *et al.* estimated species abundance in a northern temperate forest using SMA for better understanding changes in biodiversity, habitat quality, climate, and nutrient cycling [73].

### 3.2. Vegetation Index

Vegetation indices (VIs) are ‘dimensionless, radiometric relative abundance and activity of green vegetation, including LAI, percentage green cover, chlorophyll content, green biomass, and absorbed photosynthetically active radiation (APAR)’ [1]. Jensen summarized VIs benefit in maximizing sensitivity to biophysical parameters, normalizing or modeling external effects, normalizing internal effects, and assisting validation effort and quality control [1]. Additionally, VIs are simple to understand and implement, easy to quickly calculate, and useful to track temporal characteristics. To date, hundreds of VIs have been used in all kinds of applications of remote sensing. VIs can be roughly categorized into two groups, *i.e.*, biophysical indices and biochemical indices [74]. Biophysical indices represent those designed to link with vegetation biophysical characteristics including structure and condition. They can be grouped into simple ratio-based indices (e.g., Simple Ratio [SR]; [75]), soil-line-related indices (e.g., Soil Adjusted Vegetation Index [SAVI]; [76]), and chlorophyll-corrected indices (Ratio TCARI/OSAVI [Transformed Absorption in Reflectance Index/Optimized Soil Adjusted Vegetation Index]; [77]). Biochemical indices are those mainly employed to estimate vegetation biochemical properties such as Cellulose Absorption Index (CAI) and Lignin-Cellulose Absorption Index (LCAI) [78].

No doubt that NDVI is the most well-known vegetation index. Its use in EBC has been considerably reviewed by [2,13,14], *etc.* Nonetheless, other indices that are commonly used in the relevant applications are not taken seriously enough in the aforementioned review papers. For example, SR was validated to perform best in early and intermediate forest stages for the assessment of LAI based on ASTER data in East African rainforest ecosystems [79]. The modified soil adjusted vegetation index (MSAVI) was selected as the optimal vegetation index in a linear mixture model to map canopy fractional cover in tropical forests in the Amazonian state of Mato Grosso (Brazil) [80]. Haboudane *et al.* demonstrated that the existing VIs (e.g., NDVI, SAVI, Triangular Vegetation Index [TVI], and Modified Chlorophyll Absorption Ratio Index [MCARI], *etc.*) were either sensitive to chlorophyll concentration changes or affected by saturation at high LAI levels, whereas a modified triangular vegetation index (MTVI2) and a modified chlorophyll absorption ratio index (MCARI2) are proved to be the best predictors of green LAI [77]. Additionally, other recently proposed VIs such as WDRVI (Wide Dynamic Range Vegetation Index; [81]), L-ATSAVI (Litter-corrected Adjusted Transformed Soil Adjusted Vegetation Index; [74]), and VIUPD (Vegetation Index based on a Universal Pattern Decomposition; [82]). However, traditional measures such as the coefficient of determination and root mean square based on regression statistics, are not capable of evaluating the performance of VIs on the estimation of biophysical parameters because the sensitivity of a VI may change substantially with vegetation density [83]. Therefore, a statistical sensitivity function was developed to summarize the overall relationship between VIs and biophysical parameters instead of a constant [83].

### 3.3. Inversion Algorithms

Various process-oriented models are developed to characterize Earth environments because traditional methods based on simple statistical relationships are often sensor-dependent, and site-specific [84,85]. These models represent the in-depth understanding of physical processes deriving the Earth system, and are unquestionably useful in Earth observations in support of EBC [85]. Generally speaking, models can be run under two modes, namely inverse mode and forward mode. An inverse mode applies outputs to retrieve inputs that cause them, while a forward mode applies inputs to obtain resulting outputs. For example, Boyd and Danson suggested that a remote sensing model can be used to simulate the reflectance of forest canopies [84]. The forward mode treats data on the forest canopy variables as the inputs and the spectral signature as the output but the inverse mode is converse process, *i.e.*, the spectral signature is the input and estimates of the forest biophysical variables are the outputs. Obviously, the inverse model is more frequently used in remote sensing. The core of inverse model is inversion algorithms, which mostly follow the physical laws and establish cause-and-effect relationships [85]. In order to understand remote sensing signals and develop practical inversion algorithms to estimate land surface variables, physically-based models are advised to discuss the following three areas [86]: atmosphere (atmospheric radiative transfer modeling), land surface (surface radiation modeling), and sensor (sensor modeling). Liang grouped inversion algorithms into four categories: model simulation and statistical analysis, optimization algorithms, look-up table algorithms, and data assimilation [86]. Several recent examples are provided below to display the applications of inversion algorithms in EBC.

In order to monitor and model storm-water pollution, Park and Stenstrom proposed a Bayesian network approach, which falls into the category of model simulation and statistical analysis [87]. A leaf radiative transfer model called the LIBERTY (Leaf Incorporating Biochemistry Exhibiting Reflectance and Transmittance Yields) was selected and incorporated with three pigments to better understand relationships between leaf biochemical, biophysical, and spectral properties [88]. A look-up table approach was developed to estimate LAI [89]. Migliavacca *et al.* assimilated remotely sensed vegetation index time series, such as MODIS NDVI, into a process-based model BIOME-BGC (Biome-BioGeochemical Cycles) to estimate the gross primary production (GPP) of agro-forestry ecosystems [90]. However, an intrinsic problem in inverse models is the process from inputs to outputs is often non-invertible, *i.e.*, more than one combination of inputs results in the same output of spectral signature. Liang stated that, because it is still a nonlinear, ill-posed problem to inverse land surface parameters, further research is required to focus on use of regularization [86].

### 3.4. Data Fusion

Each kind of imagery has its own benefits and drawbacks, which provide great potential to fully exploit increasingly sophisticated multisource data through data fusion. For example, MODIS imagery has significant advantage in temporal resolution (one day) but is very poor in spatial resolution (250, 500 or 1,000 m) for certain applications, whereas Landsat TM imagery performed very well in spatial resolution (30 m) but with 16-day revisit. Therefore, Hilker *et al.* developed Spatial Temporal Adaptive Algorithm for Mapping Reflectance Change (STAARCH) model to fuse high spatial- (Landsat) and temporal-resolution (MODIS) for mapping of forest disturbance [91]. A general definition of remotely sensed data (image) fusion is given as ‘the combination of two or more different images to form a new image by using a certain algorithm’ [92]. Since the late 1980s when data fusion emerged as a new topic [93], several comprehensive review papers have been published to review the data fusion techniques, such as [92–95]. In general, the fusion techniques can be categorized into two classes [92]: (1) colour-related techniques, such as colour composites (RGB), intensity-hue-saturation (IHS); (2) Statistical or numerical methods, such as principal component analysis (PCA), band combinations using arithmetic operators and others. Besides typical techniques, wavelet transform, SVM (support vector machine) and ANN (artificial neural network) represent the heart of new data fusion methods (e.g., [96–98]).

Data fusion has matured into a widely used application of EBC. Pan-sharpening technique, which is to integrate a panchromatic (Pan) image with high spatial resolution and a multispectral (MS) image with high spectral resolution [94] to produce a high spatial resolution MS image, is likely to be the first data fusion method to make installing to the commercial remote sensing software such as PANSHARP module in PCI Geomatica software. For example, Wunderle *et al.* pan-sharpened SPOT-5 imagery to classify stand age of western red cedar in British Columbia (Canada) [99]. Due to the complementary nature of optical and radar imagery, their both fusion is always at the leading edge of remotely sensed data fusion [44]. Huang *et al.* estimated the quantity and quality of coarse woody debris in Yellowstone post-fire forest ecosystem from fusion of SAR and optical data [100]. Optical (Landsat-5 TM) and SAR (RADARSAT-1 Wide 1) images were fused through the combination of PCA and IHS transforms to map geomorphological and environmental sensitivity index in the Amazonian Mangrove Coast (Brazil) [101].

### 3.5. Integration of RS and GIS

RS and GIS have a complementary nature and should develop interdependently. RS routinely provides extracted information from remotely sensed data at scales ranging from local to global and the purpose of GIS is to store, analyze and visualize spatial data [102]. Although Hinton has reviewed well the combined use of remotely-sensed data and vector GIS data [103], Merchant and Narumalani claimed the integration of RS and GIS has actually become increasingly apparent since Aronoff [104,105]. Merchant and Narumalani listed key factors to benefit the integration, including development of theory and analytical methods, advances in computing (hardware and software) and global positioning system (GPS) technology [104]. A state-of-the-art definition of the integration is given as ‘the use of each technology to benefit the other, as well as the application of both technologies for modeling and decision support’ [104]. Ehlers *et al.* proposed a three-level taxonomy of the integration [106]. First-level integration happens in the level of separate but equal data exchange between GIS and image analysis systems, e.g., displaying GIS (usually vector) data and remotely sensed (raster) data simultaneously. Second-level integration permits seamless tandem or combined raster-vector processing based on a common use interface. Certain RS or GIS software has capability of performing the second-level integration. For example, the aforementioned Definiens’ Developer is capable of incorporating GIS data directly into image processing – image segmentation [69]. Third-level integration operates RS and GIS as a unified system, and finally generates an integrated model of the real world, e.g., accommodating raster and vector data in a hierarchical structure. Moreover, Gao pointed out GPS must be involved with the integration to build up seamless RS-GIS-GPS integration for geospatial information analysis [107]. Campbell, and Merchant and Narumalani summarized the contribution of RS to GIS, and GIS to RS [25,104]. The contribution of RS to GIS includes: (1) RS develops thematic layers for GIS, such as surface elevation (Digital Elevation Model [DEM]), land use and land cover mapping, biophysical parameters, feature extraction and landscape change; and (2) RS provides orthoimagery as base data, which plays key role in positioning, registration and geo-referencing. The contribution of GIS to RS consists of (1) mission planning; (2) ancillary data for geometric and radiometric correction, and image classification; and (3) collection, organization and visualization of reference data.

Foody demonstrated many commonly used examples of RS and GIS for biodiversity applications. The following review focuses on the promising applications of the integration in EBC in 2009 [102]. For example, an adaptable method integrating low-cost remote sensing imagery and GIS was developed to assess forest cover change and conversion in support of decision-makers in assessing regional and local land use and planning forest conservation measures [108]. Giriraj *et al.* applied data generated from RS and GIS to categorize habitats, and then determined the relationship between the habitat categorizations and species-distribution patterns in tropical rain forests of Southern Western Ghats (India) [109]. Dong *et al.* pointed out that the integration of high-resolution RS images and GIS technique is an effective way to analyze the landscape changes at river basin scale [110]. In the management of water resources, RS and GIS integration techniques were used to design sustainable development plan of area and locale watershed [111], river inundation impact reduction [112], rainwater harvesting for drinking [113].

## 4. Conclusions

Remote sensing plays an increasing role in EBC research, especially regarding large spatial and/or long-term temporal scales. Moreover, the use of remote sensing deepens with the support of state-of-the-art remote sensing products and technology. Certainly, it is impossible to make progress without the assistance of GIS and GPS. It is believed that remote sensing will develop in a path similar to that of computer science, which has penetrated all aspects of human life. EBC performs as a propeller to push up the naissance of advanced remote sensing instruments and techniques. For example, the object-based image analysis (OBIA) is maturing in hopes to answer the question “why are remote sensing and digital image processing still so focused on the statistical analysis of single pixels rather than on the spatial patterns they build up” raised by [114]. Blaschke summarized the status of OBIA for remote sensing through a comprehensive review several thousand abstracts [115]. However, with the popularity of remotely sensed data and commercial remote sensing packages, it is easy to obtain processed remote sensing products based on certain algorithms or modules. These products can be applied to answer questions in the field of EBC. But, it is noteworthy that these products may not be suitable or accurate enough to use. Therefore, it is still urgent to make EBC practitioners and remote sensing specialists communicate efficiently.

## Figures and Tables

**Table 1.** Hyperion Imaging Spectrometer Characteristics (adapted from [26]).

**Characteristics**	**Values**
Sensor Type	Push-broom imager
Wavelength Range	400–2,500 nm
Number of Spectral Bands	220
Spectral Resolution	10 nm
Spatial Resolution	30 m
Swath	7.5 km
Digitization	12 bits
Altitude	705 km
Repeat	16 day

**Table 2.** Disaster Monitoring Constellation (DMC) on Orbit (adapted from DMC International Imaging Ltd.).

**Designation**	**Type**	**Imager**	**Launch**	**Waveband**
Alsat-1	DMC	32m MS	2002	✓ MS
UK-DMC	DMC	32m MS	2003	NIR: 0.77–0.90 μm
Nigeriasat-1	DMC	32m MS	2003	Red: 0.63–0.69 μm
Beijing-1	DMC+4	32m MS/4m Pan	2005	Green:0.52–0.60 μm
Deimos-1	DMC	22m MS	2008	✓ Pan
UK-DMC2	DMC	22m MS	2008	0.50–0.80 μm

P.S. MS = Multispectral; Pan = Panchromatic
